# Building a Twitter Sentiment Analysis System with Recurrent Neural Networks

**DOI:** 10.3390/s21072266

**Published:** 2021-03-24

**Authors:** Sergiu Cosmin Nistor, Mircea Moca, Darie Moldovan, Delia Beatrice Oprean, Răzvan Liviu Nistor

**Affiliations:** 1 Synergy Crowds OÜ, 10141 Tallin, Estonia; sergiu.nistor@synergycrowds.io (S.C.N.); mircea.moca@synergycrowds.io (M.M.); 2 Department of Computer Science, Babeş-Bolyai University, 400084 Cluj-Napoca, Romania; 3 Business Information Systems Department, Interdisciplinary Centre for Data Science, Babeş-Bolyai University, 400083 Cluj-Napoca, Romania; 4 Coaching Consult, 400191 Cluj-Napoca, Romania; delia@edalt.institute; 5 Department of Management, Babeş-Bolyai University, 400591 Cluj-Napoca, Romania; razvan.nistor@econ.ubbcluj.ro

**Keywords:** sentiment analysis, recurrent neural network, twitter, classification, attention mechanism

## Abstract

This paper presents a sentiment analysis solution on tweets using Recurrent Neural Networks (RNNs). The method is can classifying tweets with an 80.74% accuracy rate, considering a binary task, after experimenting with 20 different design approaches. The solution integrates an attention mechanism aiming to enhance the network, with a two-way localization system: at memory cell level and at network level. We present an in-depth literature review for Twitter sentiment analysis and the building blocks that grounded the design decisions of our solution, employed as a core classification component within a sentiment indicator of the SynergyCrowds platform.

## 1. Introduction

Nowadays, social media touches all domains of the social activity. People share information in order to better understand and inform others, related to things they care for, e.g., products, services, events, or places. A global system like Twitter allows people to express their feelings in a relatively short multimedia message.

Understanding what people feel related to products or services is important both for the decision-makers that control the respective products/ services and also for their consumers. Building aggregate knowledge for decision-makers can be done in the form of indicators. Indicators are values that help decision-makers to ground their decisions.

Building indicators for measuring the sentiment of people on a specific topic of discussion is a very difficult task. Among others, it requires building a performant natural language processing (NLP) solution that is capable of extracting sentiment from sequences of characters with high accuracy.

The literature presents various models for extracting sentiment from text and more specifically from tweets. However, building a sentiment indicator for a particular set of topics of interest requires building a complete solution that is designed and validated for the particular domain of interest.

Recurrent neural networks (RNNs) are a variation of artificial neural networks that can model sequences [1]. The problem of sentiment analysis can be viewed as a sequence to value problem. The input is the text which is a sequence of tokens. These tokens can be, for example, words or characters. The output may be a single value denoting the sentiment expressed.

Modern RNNs are based on memory cells, which are able to learn dependencies between data points placed further apart in the sequence. Attention mechanisms [2] were created to enhance the ability of RNNs to find the correct dependencies between the elements in the input.

The vast majority of the papers introducing methods for sentiment analysis are using rather small datasets, as the process of manually labeling the text is very slow. The authors of [1,3] have demonstrated the importance of the dataset size for deep learning methods to show their true potential.

In this paper, we present a method for extracting sentiment from tweets based on recurrent neural networks, trained on a large corpus containing 1.5 million tweets [4]. This particular work describes the design of the proposed solution and the attained performance through a set of experiments conducted for 20 architecture design approaches. Furthermore, this work describes the integration of the proposed solution as a classification component within the execution flow of a sentiment indicator of the SynergyCrowds platform.

The contributions of this paper are as follows:

1. The design and implementation of a sentiment analysis application using deep learning methods are described and discussed.
2. Twenty different network architectures competed inside an experimental framework, aiming at identifying a practical viable solution, while setting a benchmark in terms of performance.
3. The opportunity of including an attention mechanism within the application flow was tested, in order to focus the learning process towards the most important parts of the sentences.

The remainder of this paper is organized as follows. Section 2 presents a comprehensive overview of related work, with focus on models for extracting sentiment from text, especially tweets, highlighting challenges. In Section 3, we describe the architecture and specific mechanisms of the proposed solution. Section 4 presents the experiments that we conducted for 20 design approaches of the proposed architecture and the results obtained for the considered evaluation metrics. Section 5 explains the application of the proposed solution in a real business case within the SynergyCrowds platform. Finally, Section 6 concludes and gives future work insights.

## 2. Related Work

Sentiment analysis, as a task in the natural language processing domain, aims at extracting the sentiments of the authors of a piece of text. When the text is longer, multiple sentiments may be expressed, so it is important to separate them. When multiple sentiments are expressed, these sentiments may be orientated towards different subjects, or different aspects of the subject. To successfully perform sentiment analysis in these cases, identifying the subject and the aspect is required [1].

Microblogging is an option for expressing opinions that has grown in popularity recently, especially with the apparition of social networks, which provide the perfect tools for this activity. People can express their sentiments on certain topics in short messages and their followers can quickly analyze them, in a short time span. As stated by Pak and Paroubek [5], as increasingly more people use microblogging to express their views, it becomes a valuable source of information for sentiment analysis and opinion mining.

Because Twitter has numerous users, it is a very rich source of opinions on a wide variety of topics. Messages are usually short, as a result of the limitation on the length of the message. Even though the limit was extended to 280 characters in 2018, for most of the life of this social network, tweets were allowed to consist of at most 140 characters. The great advantage is that in such a short text, there is a good chance of only having a single subject with a sentiment directed towards that subject. As reported by Boot et al. [6], the character limit increase brought only a minor increase in the length of tweets, from an average of 70 characters to 83.

As tweets add a new layer of challenges, extracting sentiment from them may prove to be a difficult task. The tweets are short texts but these messages are written using informal language and this makes them more complicated to classify with a generic text sentiment analyzer. First, there are the Twitter-specific hashtags. They are used to express short ideas present in the tweet such as topics, for example, “#sentiment”, “#opinion” or actual emotions “#happy”, “#proud”, and “#disappointed”. Hashtags are used with a wide variety of purposes. Messages may also contain numerous emoticons that can contain valuable information about sentiment and should not be ignored.

More than a decade has passed since the pioneering work of Go et al. [7], who were among some of the first researchers attempting to classify the sentiment in Twitter messages. Their approach was based on so-called noisy labels. They considered emoticons for training the machine learning algorithms, obtaining promising results and opening the way for many others with similar research interests. This read does not focus on a certain domain, but classifies the tweets across all domains, involving a very large vocabulary, the authors suggesting more targeted approaches could lead to improved results. The importance of semantics is also mentioned as a vulnerability in classification and of interest for the future work, a statement that remains partially unsolved nowadays too.

Pak and Paroubek [5] add to the literature by also considering neutral sentiments in the classification, analyzing syntactic structures for each category in order to identify common patterns.

Another difficult challenge is the language used by people when tweeting. Not only the used vocabulary contains many slang terms, but there are numerous intentional misspellings used for different purposes. These misspellings can be used to accentuate an idea, for example, “Today I am soooooo boooored...”. Other times, misspellings can show the creativity of the author, as pointed by Mohammad and Bravo-Marquez [8] with the example “happee”. Pak and Paroubek [5] were puzzled to observe that many positive tweets contained the word “whose”. After studying the dataset, they observed the word “whose” was frequently used as a misspelled version of “who is”, for example, in the tweet “dinner & jack o’lantern spectacular tonight! :) whose ready for some pumpkins??”. After creating their own corpus of tweets, they analyzed the data gathered and made some observations. Negative posts seem to contain more verbs in the past tense and they identified a small list of verbs which are frequently used in such tweets. Positive tweets contain superlative adjectives.Interjections seem to be specific to subjective tweets, as pointed out in by Barbosa and Feng [9].

The impact of hashtags was analyzed by Mohammad and Bravo-Marquez [8]. The authors state that previous work to theirs reported that hashtags did not contain relevant information for emotion classification. The intensity of emotion, on the other hand, seems to be influenced by the presence of these hashtags. The emotion intensity scores of the same tweets were analyzed, before and after the removal of hashtags. The scores differed according to the presence or absence of these features.

Kouloumpis et al. [10] use hashtags and abbreviations in order to perform a three-way classification of sentiments in tweets, in addition to the previously mentioned emoticons. The impact of intensifiers in messages (such as all caps words) was also evaluated as a feature for the classification. Other types of features served as input for the classifier, like the presence or absence of certain unigrams and bigrams that the authors found to have the highest information gains. Another feature was the presence of words that were tagged in a lexicon as being positive, negative, or neutral. The number of instances of each part of speech is also a feature candidate. Algorithm AdaBoost.MH was the used classifier.

Hashtags are also considering for clustering by Muntean et al. [11], in order to retrieve connections between different hashtags and their representations and group them into semantically batches.

Madani et al. [12] proposed a method of analyzing the sentiment expressed in tweets by combining lexicon features with fuzzy logic.

A popular choice of features used for the sentiment analysis on tweets task is n-grams. N-grams are sequences of *n* tokens that may be phonemes, syllables, or words. This feature is considered in multiple works [5,8,10].

Experiments with monograms, bigrams, and trigrams are presented by Pak and Paroubek [5], and the best results are reported to be obtained with bigrams. Naïve Bayes classifier and support vector machines were experimented with, the former being reported to perform better. The features which were given to the classifiers were the presence or absence of certain n-grams. Accuracy is increased by removing n-grams common to all classes.

Mohammad and Bravo-Marquez [8] view the problem of sentiment analysis as a regression problem. For a certain tweet a score that shows the intensity of a certain emotion is needed as an output. They created a dataset with emotion intensities for four classes. As features, they experimented with word n-grams, character n-grams, word embeddings, and features extracted from affect lexicons. For n-grams, the features were the presence or absence of certain n-grams. They report that the best results are obtained by using both word embeddings and lexicon features.

The solution described by Bollen et al. [13] begins with a preprocessing that has the purpose of extracting the terms used in the tweet. Terms are separated by white spaces, all non-alphanumeric characters are removed, all letters are converted to lowercase, and stop words are removed.

After the terms are isolated, the Profile of Mood States is used to extract sentiment from the tweets [13]. The method measures six individual dimensions of moods: tension, depression, anger, vigor, fatigue, and confusion. The authors compared their results with events taking place during the analysis and observed correlations, such as an increase in vigor during U.S. Thanksgiving Day.

Neelakandan and Paulraj [14] start their sentiment analysis method by preprocessing the tweet. This preprocessing includes removing stop words and hashtags. Multiple features like emoticon count, exclamation points count, uni-grams, bi-grams, and tri-grams are considered. The sentiment is established using a Gradient Boosted Decision Tree classifier.

Two classifiers were created for the solution proposed by Barbosa et al. [9]. The first classifier separates between subjective and objective tweets, and the second decides the polarity of the subjective ones, thus the solution outputs one of three possible categories: positive, negative, or neutral.

Murthy et al. [15] first preprocess the tweets. They remove words that are not present in dictionaries, such as slang terms, but they retain elements specific to Twitter, like emoticons, hashtags, and usernames, also correct misspelled words. For classifying the sentiment, they use a list of emoticons which were manually annotated for positive or negative sentiment and they search for these emoticons in the input tweet.

A neural network methodology consisting of experiments involving three networks was introduced by Tang et al. [16] as the Sentiment Specific Word Embedding, learning sentiments from massive distant-supervised tweets. They also use emoticons in order to collect the messages. Results were improved compared to traditional neural networks by combining the syntactic context of words with sentiment information.

As deep learning started to develop, specific solutions for NLP were also employed. A deep learning approach was proposed by Severyn and Moschitti [17] through convolutional neural networks (CNNs), stressing the importance of parameter weights initialization for the network. Briefly, their method consists of a three-step process: words embedding realized using a neural language model, the convolutional neural network refining the embeddings on a distant supervised corpus, and, finally, the parameters of the network being used to initialize another network with the same architecture, trained on a supervised corpus. The results were very promising at the time for the use of deep learning in the field of sentiment analysis for tweets.

Agarwal et al. [18] proposed an ensemble method using a convolutional neural network to extract local region information from the sentence and a recurrent neural network to capture long-term dependency information. Their aim however was not sentiment analysis, but identifying paraphrase in short text messages such as the tweets. The added value of this approach is in the field of semantics of short messages. As we already mentioned, the noisy tweets bring many problems in processing due to their specific characteristics.

When using deep learning, it became common to use word embeddings. As explained by Wehrmann et al. [19], these are a mapping from words to an n-dimensional space. The points corresponding to the words are produced in such a way that words having similar meaning will be close to each other. For example, words such as “happy” or “joyful” should be neighbors in the n-dimensional space, as their meanings are alike.

Another classifier which employs both a convolutional neural network and a recurrent neural network is presented by Stojanovski et al. [20]. The first step is a preprocessing step similar to the ones used by other methods. The authors argue that even if the pretrained word embeddings are effective at coding semantic and syntactic regularities, they are not as good at coding the sentiment expressed by the words. For this reason, during back-propagation the word embeddings are also updated. The hidden units present in the recurrent neural network used are gated recurrent units. The authors base their choice on the smaller number of learnable parameters, the potential need of less data to generalize and faster training. The output of the recurrent network is also fixed-sized. The outputs of both networks are concatenated and the final decision is taken with a fully connected softmax layer.

The problem of multilingual sentiment analysis on tweets is addressed by Wehrmann et al. [19]. The authors report that work done previous to their solution usually integrated machine translation to analyze sentiment on texts written in different languages. They argue that machine translation may introduce loss of information and a model which is able to identify the sentiment from texts written in various languages without any translation would be a superior approach. They also justify the need of a multilingual solution by pointing that there are texts written in more than one language. In the case of tweets, it is not uncommon to encounter words or expressions written in a different language than the main language of the post.

As previously mentioned, word embeddings are a common tool used together with deep learning approaches. In a multilingual context, word embeddings may seem to make sense as a solution because words with the same meaning will be close to each other, even if they do not belong to the same language. The problem is that by adding words from different languages, the size of the dictionary explodes.

The solution proposed by Wehrmann et al. [19] is the usage of character embeddings. Each considered character is converted into a one-hot vector. For embedding the words, a convolutional neural network is used. Only one convolutional layer with rectified linear unit (ReLU) activation is present in the architecture proposed by them. The authors claim that this layer learns relationships between characters and words and is even able to correct typos. The next layer of the network is a max-pooling-over-time, similar to the one presented by Stojanovski et al. [20].

An update is proposed by Alharbi et al. [21], where user behavioral information is added for analysis on the form of other features obtained from Twitter. These features are either directly available, like the number of a user’s tweets, number of tweets posted by a user, number of followers, etc., or calculated by the authors, such as number of positive/negative/neutral tweets posted by a user and number of adjectives/nouns/verbs/etc. The results showed some improvements in accuracy, compared to other (more) traditional methods, even on unbalanced datasets. We consider the work important in opening this area to the idea of prescriptive analytics, where the impact of a tweet could be assessed, based on the sentiment analysis and behavioral data. An interesting approach [22] in this respect, introduces the sentiment trajectory concept by analyzing the location of users sending Twitter messages and combining them to different events. The system uses real-time data in order to predict users’ sentimental paths.

A versatile framework is presented by Abid et al. [23] by combining a convolutional neural network and a recurrent neural network aiming to perform on small, medium, or large datasets. The architecture consists of four major layers: Word embedding using random initialization and unsupervised learning, RNN for the long-term dependencies, CNN with several filters in place, and the Global Average Pooling Layer. Apart from obtaining a model with higher accuracy compared to other base models, the authors were also concerned about keeping the computational cost in reasonable limits, the reason for combining CNNs with RNNs. The results obtained suggest this architecture can perform efficiently and also reduce the number of parameters involved in the neural network (NN).

An ensemble deep learning methodology was introduced by Kotteti et al. [24], who proposed a majority voting system where several neural networks are trained on time-series data in order to predict rumors.

Seo et al. [3] created a benchmark analysis for different deep learning methods, using 13 datasets. They conclude that performance is dependent on the dataset size, which is typical for deep learning algorithms. Furthermore, they show how the complexity of the network helps in improving the results for the RNNs, but not for the CNNs.

Because of different aspects of the semantics, messages can include multiple sentiments inside, becoming difficult for a classifier to differentiate them. This difficulty was tackled by a method called attention mechanism, which can indicate the model to weight more on the important part of the sentence. Wang et al. [25] introduced an attention-based long short-term memory network in order to focus on the aspect-level sentiment classification, managing to only slightly improve the benchmark models.

Similar experiments were conducted by Tang et al. [26], who used a neural attention model over external memory, in order to focus on the aspect level sentiment classification. A comparable work, but tested on Twitter data, was done by Chen et al. [27], who introduced a framework using a multiple-attention mechanism for capturing sentiment features separated by a long distance, aiming for more robust results against irrelevant information. Note that even if the presented results overpassed the base models, the improvements seemed minor. Moreover, probably due to noisy data, the accuracy results on the Twitter dataset were significantly inferior to datasets obtained from other sources.

Xing et al. [28] proposed a solution for twitter sentiment analysis which integrates both a CNN and an attention mechanism. Word embeddings are passed through an attention-based input layer and then a CNN is used to analyze the sentiment that is expressed.

A multiconvolution and pooling method for classifiying text sentiment was introduced by Dong et al. [29]. Their approach was to add four convolution operations in the word embedding dimension and adding average pooling to extract more detailed features. The method was tested on English and Chinese datasets. The best results in terms of classification improvement are shown to be on the Chinese sentiment analysis experiments.

Results of applying both traditional ML methods and deep learning methods on a Lithuanian language dataset are provided by [30]. Their findings cannot conclude which of the two group methods are the best, but this might be due to the relative small data (about 10,000 instances).

We identified several approaches on analyzing sentiment based on the same Twitter messages corpus [4] as our research and used them as benchmarks for the results, in Section 4. Caragea et al. [31] used the dataset in order to train different deep learning algorithms for predicting optimism/pessimism. In their experimental setup, the authors tested the efficiency of the models on a different dataset, also containing a neutral class. Another research, by Appel et al. [32], focuses on building a hybrid method consisting of NLP techniques, a lexicon, and fuzzy set techniques to prove it could perform better than isolated ML methods. The same data were used to train a CNN with a single layer convolution in [33], with an interest mainly on the tuning of the neural network, without comparing the results to other architectures. Yusuf et al. [34] are using classical NNs to extract sentiments from tweets. Their experiments implied several datasets (including the same corpus as ours) and different training/testing combinations among them.

## 3. Proposed Solution

In this section, we describe the solution that we built considering the principles and mechanisms presented in Section 2. First, we present the overall execution flow, then the architecture of the recurrent neural network—as this is the technical foundation of our solution, then we discuss the attention mechanism that we attached in the aim of improving the performance of the proposed network.

### 3.1. Execution Flow and Design Considerations

**Figure 1:**
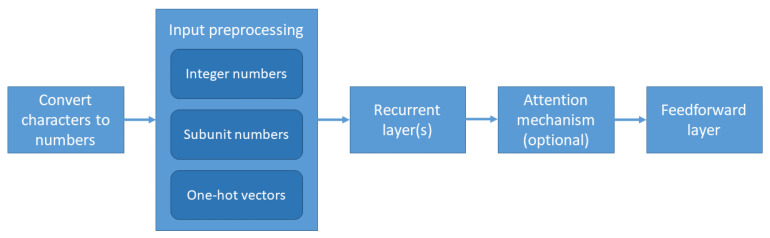
Pipeline of proposed solution.

Our solution first converts the text into a numerical representation. Afterwards, preprocessing is applied on the input. The information is passed to one or more recurrent layers for processing. Optionally, an attention mechanism is then applied. The final classification is done by a feedforward layer, which outputs the scores for each considered sentiment, positive or negative, the larger score deciding the class. Figure 1 presents the pipeline of our solution.

The input text is processed character-by-character and no part of the tweet text is filtered out. This decision was made to take into account all available information when analyzing the tweet. Other works [5,10] use a dictionary of words. By processing only alphanumeric characters and grouping them into words, information like emoticons, emojis, and hashtags is lost. Even if these features are treated separately, by using a predefined dictionary of words, creatively spelled words would be lost because they are not in the dictionary, or they would require to be corrected, which could introduce errors in the text and distort the result.

The vocabulary that we use contains letters, digits, punctuation marks, and emojis. We defined the vocabulary based on the characteristics of the dataset that we used in our experiments. The frequency of each character was computed and a threshold was applied to select the considered characters. Using the vocabulary, we defined a dictionary which maps each character to a numerical value.

There are situations when the input text contains characters that are not present in the defined dictionary. For treating such situations, we introduced an “unknown” character in the dictionary.

For our solution we use one of three input text preprocessing techniques: (1) Integer Numbers, (2) Subunit Numbers, and (3) One-hot Vectors. Integer Numbers is converting each input character into an integer number according to the character dictionary. The second preprocessing also involves normalization. Subunit Numbers convert each character into a subunit number by dividing the integer value of the character by the size of the vocabulary. One-hot vectors are a specific approach to RNNs. Each character is converted into a vector which has zeros on all positions excepting the position corresponding to the character, on which a one is present.

Table 1 shows an example of preprocessing the text “SENTIMENT”. The used dictionary is {S:0, E:1, N:2, T:3, I:4, M:5}. For the one-hot vector representation, each letter is assigned a column in Table 1. In each such column-vector, only one position is set to 1, i.e., the position corresponding to the letter in the dictionary.

### 3.2. Recurrent Neural Networks

For building a solution to finding the sentiment expressed in a tweet, we built and experimented with 20 different recurrent neural network architectures. We used a large set of annotated tweets for our experiments, dataset which is described in Section 4.2 and in Table 2. The architectures that we used are defined in Table 3. We chose this type of networks due to their ability to process sequences [35]. RNNs make use of sequential information by making the same computations for each sample, but taking into consideration previous computations [36,37]. Furthermore, RNNs keep an internal state which is updated after processing each point in the sequence. At each processing, the value of the internal state is also taken into consideration.

**Figure 2:**
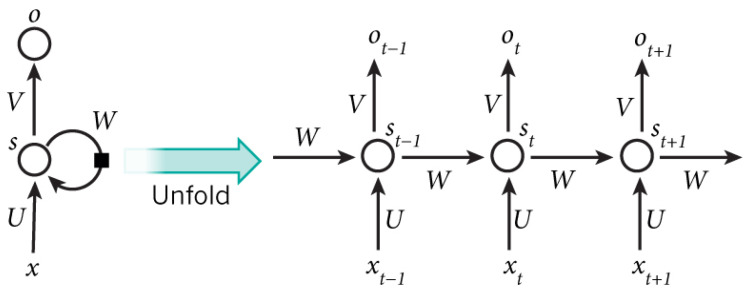
Example of recurrent neural network (RNN) [37] and its unfolded version (without recurrences) in three time steps.

In the left side of Figure 2, we give an example of an RNN representation. The graph representation of an RNN differs from the one of a feedforward network, being more difficult to understand. The difference is that the RNN contains recurrent edges, which can form cycles in the representation, such as depicted on the left side in Figure 2. In reality, these edges connect computations at different time steps and cannot form cycles inside a single time step that is using conventional edges [35].

Backpropagation through time (BPTT) is used to train RNNs. First, the network is unfolded and is transformed into a feedforward network. The unfolding process is graphically represented in Figure 2. The number of layers of the unfolded network is equal to the number of time steps that are processed, and each layer uses the same learnable parameters, so parameter sharing is employed. In this new representation, backpropagation can be used to train the network.

Earlier recurrent neural networks had the problems of exploding or vanishing gradients. These problems prevented them from being able to learn dependencies between data points that were at larger distances in the sequence. The solution came in the form of long short-term memory (LSTM) cells [38]. These cells have a so-called “constant error carousel” (CEC) which is a recurrent edge with a weight of one. In this way, vanishing and exploding gradients are avoided. The LSTM cell is a group of operations which replace the hidden units of a recurrent neural network. We considered LSTM as one of the approaches for designing our network.

**Figure 3:**
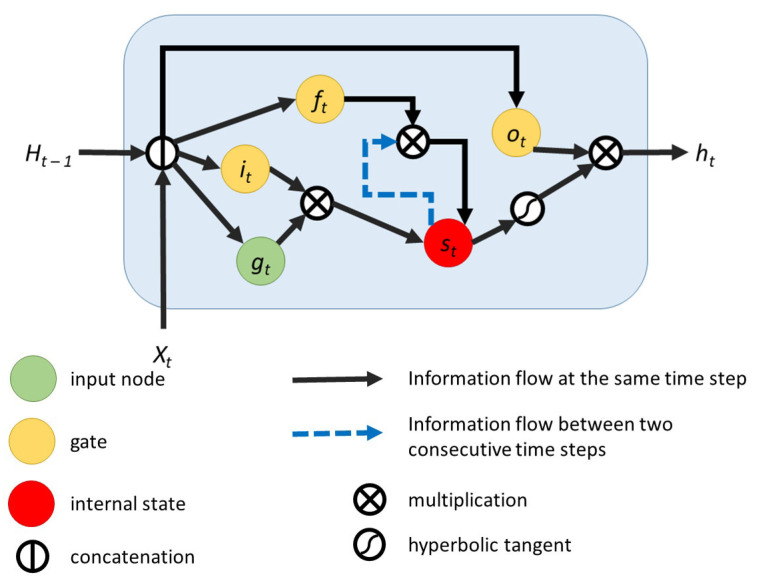
Long short-term memory cell.

Figure 3 depicts a graphical representation of this memory cell. $X_{t}$ denotes the input of the cell at the current time step, $H_{t-1}$ denotes the hidden states of all the cells at the previous time step, and $h_{t}$ denotes the hidden state of the cell at the current time step. LSTMs have gates which control the flow of information. The input gate controls how much information is let to enter the cell, the forget gate helps the cell to flush the contents of the internal state, and the output gate controls the information that leaves the cell.

**Figure 4:**
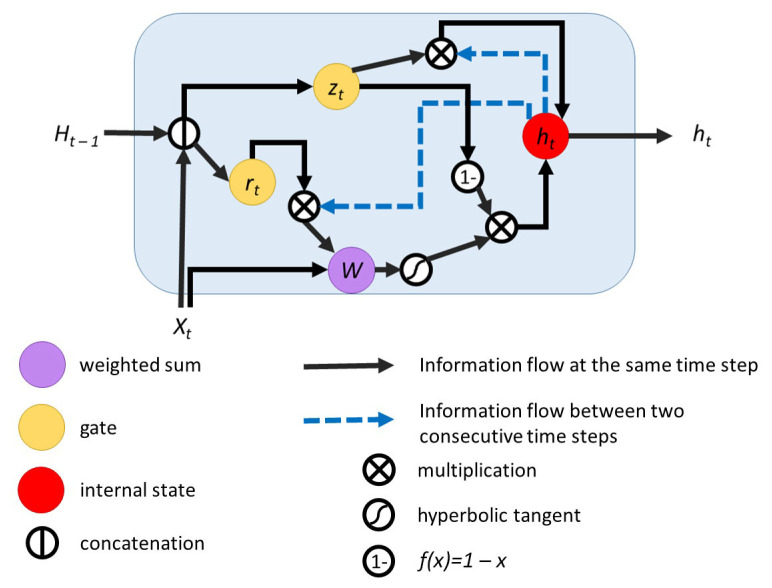
Gated recurrent unit.

As the LSTM cell was successfully used in numerous applications, variations to it were proposed. The gated recurrent unit (GRU) [39] is a simplified version of the LSTM. It only uses two gates and has less learnable parameters. This design was used to obtain performant solutions and it has been growing increasingly popular [40]. The graphical representation of this cell design can be seen in Figure 4. As it can be seen, the hidden state and the internal state coincide for GRU.

For our solution, we also considered bidirectional recurrent neural networks. This design principle of the network architecture is two layers of artificial neurons: one having recurrent connections to the computations of previous data points from the sequence and the other having recurrent connections to the computations of the next data points of the sequence.

**Figure 5:**
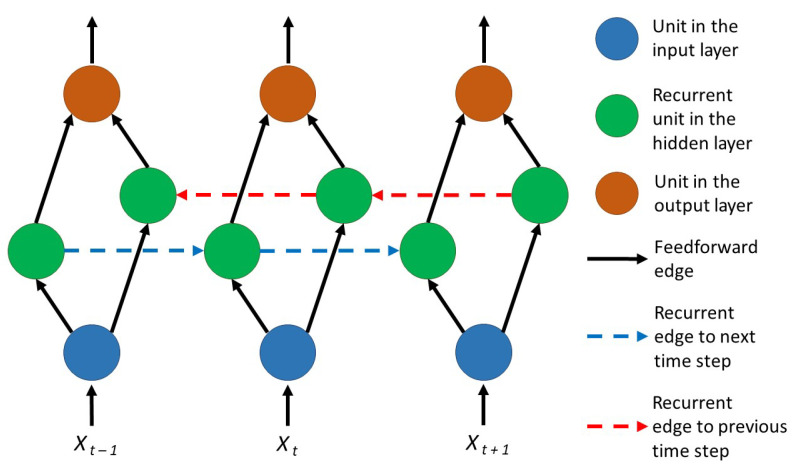
Bidirectional recurrent neural network.

Figure 5 shows the graphical representation of a bidirectional recurrent neural network. This design approach successfully allows the use of memory cells. RNN architectures can be bidirectional, and LSTMs, GRUs, or other memory cells can be used as hidden units.

We used the design approaches that we previously described as defined in Table 3 and the results for experimenting with these mechanisms are presented in Section 4.5.

### 3.3. Attention Mechanism

In this subsection, we describe the attention mechanism which we used in our solution. We present the context in which this mechanism was introduced, how our problem is different, and what modifications we made to adapt the attention mechanism to our method.

An attention mechanism was proposed in by Bahdanau et al. [41] for enhancing recurrent neural networks. The purpose was to create a better neural machine translation algorithm. The classical approach when using RNNs for machine translation was to use an encoder RNN which creates a fixed-sized vector which contains all information about the input sentence. The decoder RNN takes this vector and outputs the translation. It is argued that this fixed-sized vector has a limited representational power and this prevents the encoder–decoder architecture from accurately translating longer sentences [41].

The proposed solution is to avoid using a fixed-sized representation [41]. The encoder is a bidirectional RNN. For each word in the sentence, the encoder outputs an annotation which summarizes the information around the word. These annotations are then processed by the decoder, and not just a fixed-sized vector.

The attention mechanism is attached to the decoder [41]. Using it, the decoder is able to focus on relevant parts of the input sentence when predicting each word of the output sentence. For emitting each word, the decoder receives as input a context vector. Because the decoder is a RNN, it also has a hidden state which helps it “remember” what was already predicted. This context vector is a weighted sum of the annotations produced by the encoder. The weight is computed as the softmax applied on scores produced using what is called “alignment model” [41]. This alignment model is a feedforward neural network that takes as input for each word the annotation and the hidden state of the decoder.

We integrated an attention mechanism—similar to Bahdanau et al. [41], in our sentiment analysis recurrent neural network which we denote by ATT. Previously to the addition of this mechanism, the prediction of the sentiment expressed in the text was created by passing the output of the RNN for the last word through a feedforward layer. This may be a limitation if longer texts are considered. Even if tweets are short texts, an attention mechanism might help our model and that is why we experimented with such an approach.

Our implementation of the attention mechanism uses a feedforward layer which takes as input the output of the RNN for each word. In the solution proposed by Bahdanau et al. [41], the state of the decoder was also given as input, but our model does not contain a decoder as the problem of sentiment analysis, in our formulation, is not a sequence to sequence problem, but a sequence to value problem. The scores produced by this layer are then processed using a softmax function and the resulting values are used as weights.

We create our context vector by multiplying each annotation produced by the RNN with its corresponding weight and then summing up these vectors. The final context vector is then passed through a feedforward layer to produce the class scores. This approach allowed us to use both unidirectional RNNs, but also bidirectional RNNs. The bidirectional architectures can produce annotations that summarize the context around a word, considering both directions, not only what the sequence contains before the word [41].

**Figure 6:**
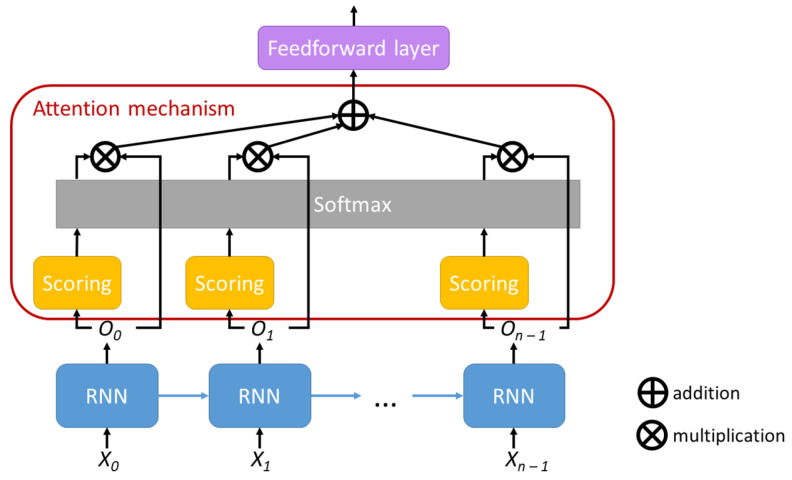
Attention mechanism designed at network level.

Figure 6 shows a graphical representation of the attention mechanism designed at network level. $X_{t}$ denotes the input to the RNN and $O_{t}$ denotes the output of the RNN at time step *t*. The output is processed by the “Score” module which produces the annotation for each value in the sequence. The annotations are passed through a softmax layer and afterwards each annotation is multiplied by the corresponding RNN output. The results of the multiplications are summed to produce the context vector, which is passed through the feedforward layer to produce the class scores. When this attention mechanism is not used in our solution, the output of the RNN produced after the last element in the sequence, $O_{n-1}$ in Figure 6, is passed directly to the feedforward layer.

In our solution, we also considered the extension to the LSTM cell as defined by Cheng et al. [42] and which we denote by LSTMN. The respective approach replaces the cell with a LSTM network. This design has integrated memory tapes and attention mechanisms. The network contains a hidden state tape which stores data used to interact with the environment and a memory tape. The memory tape is used to help the RNN better memorize the sequences that it processes. When a new token is processed, attention is used to read what is already in the memory and link it to the token. Afterwards, information about the token is stored in the memory tape. As described by Cheng et al. [42], the memory increases as more tokens are processed, until it reaches a certain memory span.

LSTMN is similar to LSTM, except for the internal state, which is replaced by the memory module. When the internal state must be read, the memory module computes the value based on the memory tapes. When the internal state should be updated, the module stores the new information on the tapes. As the design was also applied on sentiment analysis [42], even if not on tweets, we included it in our tweet sentiment analysis experiments.

## 4. Experiments

### 4.1. Experimental Setup

In our experiments, we used different RNN architectures to find a solution for sentiment analysis and improve its performance. We experimented with layers of LSTMs or of GRUs. We adjusted the number of hidden units per layer, as this could help the model learn more patterns. We also tried architectures with multiple layers because we expected this to help the network learn concepts of higher complexity. We also checked the impact of attention mechanisms on the performance of the model.

We store the samples in text files (CSV) which have a fixed structure. Each line of the file is a sample. The first character of the file is an integer number, more exactly either 0 or 1. This number is the sentiment annotation, 1 being positive and 0 negative. The annotation is followed by a comma and then by the text of the tweet.

When training the network, batches of 32 tweets are given at each training step. After every 100 training steps, the model is evaluated on a batch of 32 tweets from the testing set. The mean accuracy and loss on the test batch are saved and we plot these values to visualize the evolution of the network.

### 4.2. Training Data

In this subsection, we describe the dataset that we used for training and testing our network, Twitter Sentiment Analysis Training Corpus [4], as it is. This is by far the largest dataset we found in integrum, containing 1,578,627 annotated tweets. We used 80% of the samples to create the dataset on which our models were trained and the rest for testing.

This dataset is representative, as it contains not only text composed by only words and numbers, but also punctuation marks which form emoticons, hashtags, and emojis.

**Figure 7:**
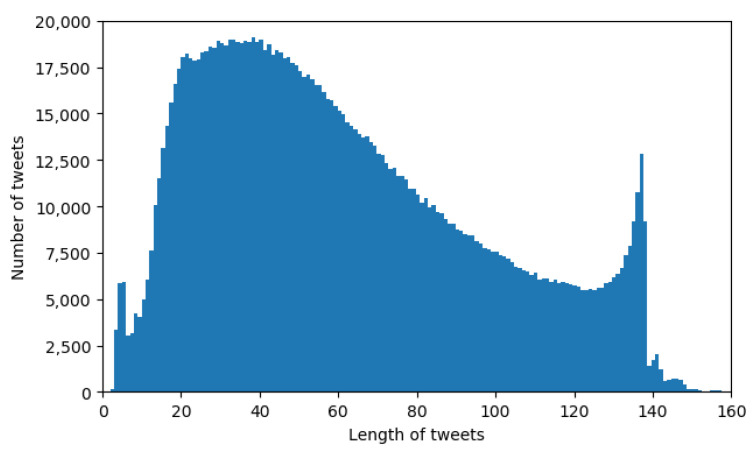
Histogram with lengths of the tweets of the dataset.

Figure 7 shows a histogram of the length of tweets contained by the considered dataset. The dataset contains even longer tweets than shown in the histogram, but in insignificant number, so we did not plot them.

Previously, Twitter had a character limit of 140 for their tweets, which was extended. The effects of the old limitation can be seen in the histogram, as most tweets do not exceed 140 characters, and we can see that the number of samples in the last bins is increasing, as the authors of the tweets were constrained to shorten their message.

We present in Table 2 the structure of the dataset. The “Percentage” line presents the percentages of data used for training and for testing out of the total, while the “Percentage” column presents the percentages of positive and negative samples out of the total. As it can be seen, the dataset is balanced with respect to the number of samples for each class.

### 4.3. Experimental RNN Architectures

As our mission is to build a performant network and we had more design options, we conducted experiments to explore the different architectural decisions. As approach for defining the experiments, we considered the most important choices when constructing a tweet sentiment analysis method based on recurrent neural networks. In each type of experiment we isolated a design decision to see how it affects the performance of the model. Therefore, in each experiment we vary only the parameters of the analyzed design decision.

The tables that are presented in this subsection describe the architecture and the metrics for each architecture. The first column indicates the architecture number, denoted with $Arch_{i}$. This is simply a codification of the architecture design approach. The same number may appear in multiple tables. This is because it refers to the same model, even if the tables may contain only the parameters that are relevant for the experiment.

In Table 3, we list all considered architecture design approaches, with their definitions. The definitions contain the number of layers, the number of units per layer, the memory cell, the preprocessing, and, optionally, if the networks are bidirectional or employ attention mechanism at network level.

### 4.4. Evaluation

For evaluating the performance of the considered architecture approaches we used the following metrics: accuracy, precision, recall, and true negative rate (TNR).

For defining these metrics, we need to first define the following particular terms.

The number of true positives (TP) is the number of samples which are labeled as positive by our model and are also annotated as positive by the creators of the dataset. The number of true negatives (TN) is the number of samples labeled as negative and annotated as negative. The number of false positives (FP) is the number of samples labeled as positive, but annotated as negative. Finally, the number of false negatives (FN) is the number of samples which are labeled as negative, but are annotated as positive. In the case of our binary classification task, TP and FP refer to the positive class, while TN and FN refer to the negative class.However, in the general *N*-class scenario, these terms can refer to any of the classes.

Equations (1)–(5) define the metrics. The ratios are multiplied by 100 to present the results in percentages.

The accuracy (Acc) is the percentage of samples which were correctly labeled by our model out of the total number of evaluated samples.

(1) $$\mathrm{Acc}=\frac{\mathrm{TP}+\mathrm{TN}}{\mathrm{TP}+\mathrm{TN}+\mathrm{FP}+\mathrm{FN}}\cdot 100$$

The precision for a given class *C* is the percentage of samples correctly classified as *C* out of the total number of samples labeled with *C* by the classifier. For the positive class, the precision is give by Equation (2) (PrecP), and for the negative class, by Equation (3) (PrecN).

(2) $$\mathrm{PrecP}=\frac{\mathrm{TP}}{\mathrm{TP}+\mathrm{FP}}\cdot 100$$

(3) $$\mathrm{PrecN}=\frac{\mathrm{TN}}{\mathrm{TN}+\mathrm{FN}}\cdot 100$$

The recall (Rec) is the percentage of samples which were correctly classified as positive out of all the samples which were labeled with this class in the ground-truth.

(4) $$\mathrm{Rec}=\frac{\mathrm{TP}}{\mathrm{TP}+\mathrm{FN}}\cdot 100$$

The TNR is the percentage of samples which were correctly classified as negative out of all the samples which were labeled with this class in the ground-truth. For a binary task, the TNR acts like a recall for the negative class.

(5) $$\mathrm{TNR}=\frac{\mathrm{TN}}{\mathrm{TN}+\mathrm{FP}}\cdot 100$$

**Figure 8:**
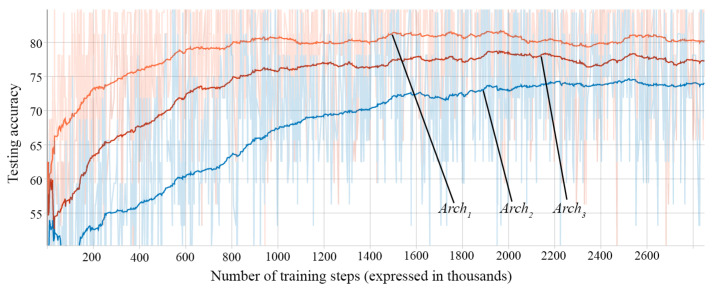
Test accuracies according to input preprocessing type: $Arch_{1}$—One-hot Vectors (orange), $Arch_{2}$—Subunit Numbers (blue), and $Arch_{3}$—Integer Numbers (red).

**Figure 9:**
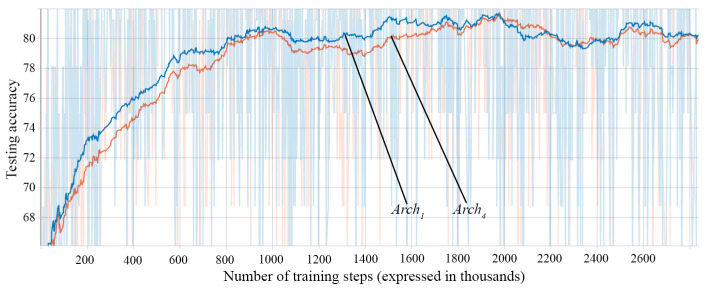
Test accuracies according to memory cells: $Arch_{1}$—GRU (blue) and $Arch_{4}$—LSTM (orange).

**Figure 10:**
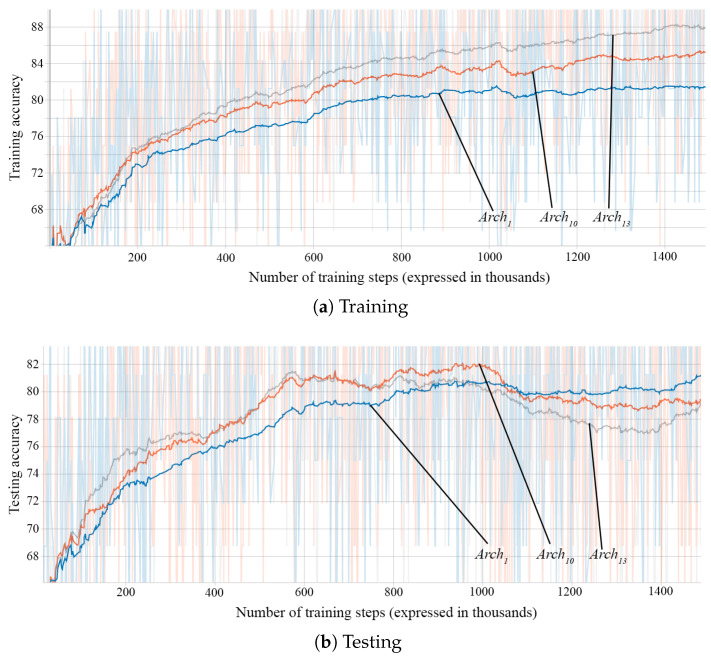
Accuracies according to the number of hidden layers: $Arch_{1}$—1 (blue), $Arch_{10}$—2 (orange), and $Arch_{13}$—3 (gray); (**a**) training accuracies and (**b**) testing accuracies.

During the training of each architecture approach, after every 100 training steps, we evaluate the model on a batch of 32 samples from the testing dataset. We store the accuracies obtained in these evaluations and plot them to visualize the progress during training. Examples of such plots can be seen in Figure 8, Figure 9 and Figure 10.

### 4.5. Experiments and Results

The first important decision is the input preprocessing type. Figure 8 shows the accuracy on the test data of three architectures ($Arch_{1}$, $Arch_{2}$, and $Arch_{3}$) of RNN at different training steps. The architectures differ between them only through the input preprocessing mechanism. The results of the fully trained networks can be seen in Table 4.

It can be observed that the one-hot approach obtains the highest accuracy and achieves better accuracy than the others in a smaller number of steps. Integer Numbers obtains better accuracy then Subunit Numbers and it is also a faster learner. The vectorial representation of the characters helps the network better distinguish the input characters due to the marking of the character on the corresponding position. This representation obtains the best results, but the drawback is the necessary memory. While the memory requirements of the other preprocessing options only depend on the length of the input sequence, the one-hot vector representation also depends on the size of the character dictionary, so the characters which are included in the dictionary must be carefully selected.

We evaluated architectures based on GRU cells or LSTM cells. The evolution of the accuracy of two such architectures can be seen in Figure 9. The two architectures for which the plot was created contain one layer of 128 units each. One uses LSTM cells and the other GRU cells.

As it can be seen in Figure 9 and Table 5, the type of memory cell does not seem to influence the performance of the network significantly. Table 5 presents the results of the two architectures. From the table we observe that the metrics for the two networks are very similar. The GRU network obtains slightly better precision for the positive class and TNR, while the LSTM network is slightly superior according to the accuracy, recall, and precision for the negative class.

We also experimented with architectures with multiple layers. We considered that two layers of units might improve performance while the number of learnable parameters is kept small enough to avoid overfitting problems. The results of the networks used in this experiment can be seen in Table 6.

The architecture having two layers of 32 memory cells, $Arch_{8}$, does not improve over the architecture with a single layer of 32 units, $Arch_{5}$. It does not improve over the 1-layered architecture with 64 units, $Arch_{6}$. This second comparison is interesting as the number of memory cells used is the same, but they are used in different numbers of layers. We expected multiple layers to perform better due to the ability of learning more complex concepts.

The 64-unit 2-layered architecture, $Arch_{9}$, performs slightly better than the corresponding networks with 1 layer with the same layer size, $Arch_{6}$, or the same total number of units on all layers, $Arch_{1}$. However, increasing the number of layers of 64 units even more to 3 ($Arch_{12}$), 4 ($Arch_{15}$), 5 ($Arch_{16}$), or even 8 ($Arch_{17}$) does not produce improvements. We observed the same lack of performance improvement when increasing the number of layers of 128 units ($Arch_{1}$, $Arch_{10}$, and $Arch_{13}$). For the case of networks with 32 units per layer ($Arch_{5}$, $Arch_{8}$, $Arch_{11}$, and $Arch_{14}$) a very slight improvement exists, as it can be seen in Table 6.

Figure 10 shows the training and testing accuracies of three networks with different numbers of layers, $Arch_{1}$ with 1 layer, $Arch_{10}$ with 2 layers, and $Arch_{13}$ with 3 layers. The networks have an equal number of hidden units per layer. We observe that the networks with more hidden layers are able to achieve better accuracies in a shorter number of steps and achieve higher final accuracy on the training dataset. However, the final accuracies on the testing dataset are similar, even slightly lower for the networks with more layers: 79.81%, 78.97%, and 77.84%. This seems to indicate that the added layer makes the network better able to learn, but it is unable to generalize well. The testing accuracies of the networks with a greater number of layers even decrease after a number of training steps, showing that they are more prone to overfitting the training data.

Table 7 gives the performance for varying the number of layers on both training and testing datasets.

The next experiment involved attention mechanisms. We experimented with the LSTMN cell design described in Section 3.3 and with the network-level attention implementation described in Section 3.3. $Arch_{4}$ from Table 5 is repeated in Table 8 for easier visualization of the comparison between this architecture that uses LSTM with no attention mechanism and the architectures which use attention.

Table 8 contains the metrics for the networks that employ attention mechanisms. Descriptions which contain ATT use our implementation of the attention mechanism at the network level. “bi-RNN” in the description means that the RNN is bidirectional (all models without this specification are unidirectional).

The addition of attention in different forms, for unidirectional or bidirectional RNNs, slightly lowers the metrics. This can be attributed to the addition of new learnable parameters. Without increasing the number of training samples, this could lead to overfitting problems.

As discussed in Section 3.3, the attention mechanism may help the model to focus on the appropriate part of the input when sequences are very long. However, from the obtained results we observe that tweets are short enough sequences, even when processed character by character, which can be processed by RNNs without the help of attention.

For the samples in the considered dataset, we observed that using the attention mechanism brings a slight improvement only. Therefore, this mechanism may have a more relevant impact when processing longer tweets or other textual sequences—like longer posts on other social networks.

Comparing the obtained values, we observe that LSTMN slightly improves precision for the positive class and TNR over LSTM and has a slightly weaker performance in terms of accuracy, recall, and precision for the negative class.

To better observe the capabilities of each considered architecture, we required a large dataset to work on. As mentioned, Twitter Sentiment Analysis Training Corpus [4] is by far the largest dataset that we found. The disadvantage of this corpus is that it has no standardized training–testing split, so it is difficult to perform comparisons between different approaches in the literature that used it. We included, for comparability, in Table 9 the results provided by other papers using the same dataset.

The author of the dataset [4] reports that a Naïve Bayes classification algorithm applied on the dataset obtained an accuracy of 75%, without mentioning which are the training and testing splits. Our algorithm surpasses this approach if we consider only the accuracy by more than 5%, but it is difficult to compare our accuracy with the reported one, as the testing sets might be different. Our approach proved to surpass, considering the accuracy, other methods tested on the same dataset, with one exception. Yet, given the very small sample used in [32] (only 2000 tweets) to obtain an accuracy of 86.55%, we can call the mentioned result as inconclusive.

## 5. Application

In this section, we describe how we use in practice the results of the work presented in this paper. The insights gained from this work grounded the design solution for the classification component of a sentiment indicator, within the SynergyCrowds platform.

This platform produces knowledge for making informed decisions when using cryptocurrencies. Such knowledge is produced as indicators and one of the calculated indicators is a sentiment indicator on cryptocurrencies.

**Figure 11:**
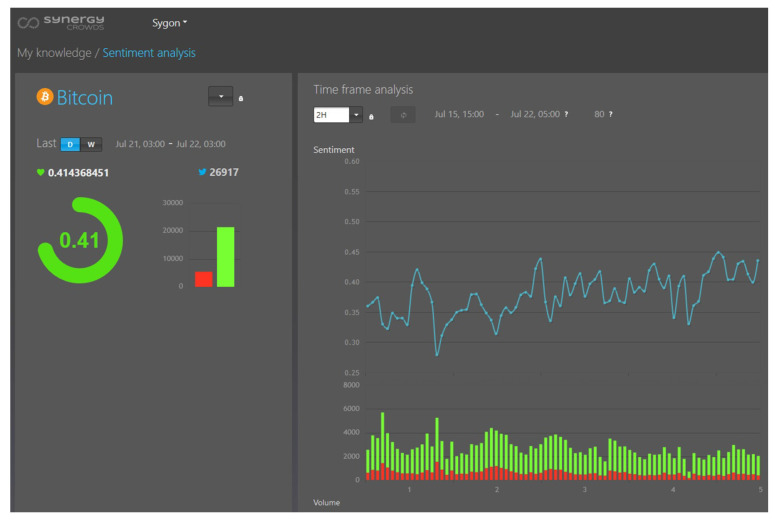
Sentiment indicator GUI within SynergyCrowds platform.

The SynergyCrowds platform provides to its users a sentiment indicator on crypto tweets. The indicator is computed per digital asset (as for Bitcoin, Ethereum, etc.) and it is delivered as a number in the [−1; 1] interval, per time frame. A value that tends to −1 indicates a negative sentiment towards the asset and a value that tends to 1 indicates a positive sentiment towards the asset (Figure 11).

The indicator detects the positive/negative sentiments, as described in Section 3. Furthermore, the indicator is computed for various time frame granularities: 1 h, 2 h, 4 h, 6 h, 1 d, 2 d, 1 w.

**Figure 12:**
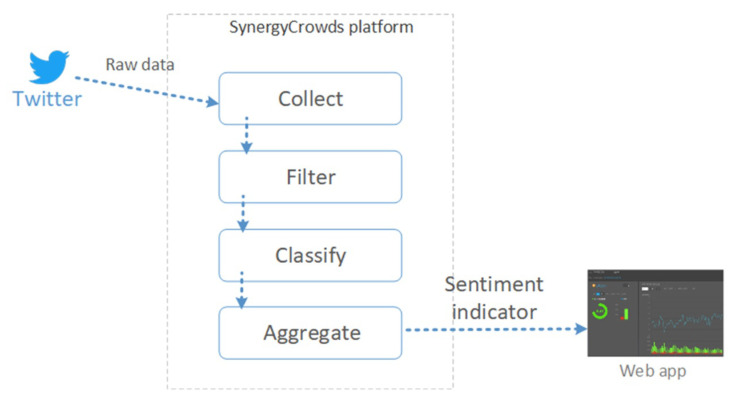
Execution flow of the sentiment indicator.

In the following, we describe the flow to compute the sentiment indicator within the platform Figure 12 presents this flow and the delivering of the indicator to an external web application.

Therefore, the execution flow starts with collecting raw data for a particular asset. These data represent tweets as returned by the API in response to the query configured for the respective asset. Then, raw data are filtered in order to eliminate tweets that do not refer strictly to the targeted asset and could not be filtered out by the query configuration.

The flow continues with the classification phase of the filtered tweets. This is performed by a classification component equipped with the $Arch_{1}$ RNN. In this phase, each tweet is assigned a sentiment score in the [−1, 1] interval.

The flow ends with computing the sentiment indicator value for various time frames, as the average of the score values of the tweets that fall within the time frames.

From a business perspective, the sentiment indicator can be further analyzed in conjunction with other time series in order to further discover knowledge like correlations. This indicator along with other additional insights can be used in portfolio management activities.

## 6. Final Conclusions and Future Work

The increase in popularity of social networks created an environment for microblogging in which people can express ideas and opinions on any topic and their posts can reach a very large number of followers. Twitter is one of the most popular social networks and a very good source of data for opinion mining.

Sentiment analysis of tweets is similar to the analysis of generic texts, but problem-specific constraints provide the opportunity of finding new and creative solutions for this particular form of writings. Many solutions were proposed due to high applicability of extracting sentiments from tweets.

We created a sentiment analysis on tweets method. We described the building blocks that we used and presented the results of our experiments. We concluded that the best preprocessing is, by far, the use of one-hot vectors. We observed that there is no significant difference in the results when using LSTM or GRU, the two most popular memory cell designs. The attention mechanisms that we employed at memory cell level or at the network level produced insignificant improvements to our method.

Our experiments involving the number of hidden layers and the number of units per layer indicated that the best number of hidden units is 128. What we found interesting is that the distribution of these units does not need to be fixed. A network consisting of one layer of 128 units performs similarly to one consisting of two layers of 64 units, or one consisting of four layers of 32 units. We observed that increasing the number of layers but keeping fixed the number of units per layer increased the performance of the model on the training set, but not on the testing set. This demonstrated that increasing the size of the network, beyond the best-performing size that we discovered, does not lead to better performances outside of the data presented to the model during training.

The domain of cryptocurrencies has greatly grown in popularity and more and more people understand the opportunities created and the great value that the domain adds. The rise in adoption means more people express their opinions towards different cryptocurrencies and crypto projects and finding the public opinion has great advantages.

For these reasons, we decided to use the method of sentiment analysis on tweets that we created to compute a sentiment indicator on these topics. This indicator shows the evolution of the opinions expressed on Twitter and can be further used to make informed decisions in the crypto space.

We experimented with many architectural decisions, but we kept the recurrent neural network design and the basic cells that already exist in the literature. A more extensive study would consider other recurrent cells or even other algorithms to observe how they compare. Furthermore, there is always the possibility of an existing dataset bias. For our current work, we limited this possibility by using a large dataset, and we observed that even though this set contains no tweets related to the cryptocurrency domain, the model trained on this corpus obtains good results on this domain, and so demonstrates generality. For a future study, we plan on using multiple datasets for our experiments so that the dataset bias possibility can be reduced even further.

We currently work to improve the design of the memory cell used in the recurrent neural network. We obtained 80.39% and 80.74% accuracies using the classical LSTM and GRU, respectively, but we expect that a unit specifically designed for our task should be able to better extract the sentiment. Nevertheless, compared to other papers using this dataset as a corpus for sentiment analysis, our work provides a clear experimental design, setting a new benchmark for future developments.

Based on a dataset that is currently built by the SynergyCrowds project, we will train our solution on crypto tweets only, in the aim of improving the performance of the classification method even further and bring more relevance to the crypto domain.

## Figures and Tables

**Table 1:** Preprocessing results for an example containing the input text “SENTIMENT”.

Preprocessing Type	Input String								
	S	E	N	T	I	M	E	N	T
Integer numbers	0	1	2	3	4	5	1	2	3
Subunit numbers	0	0.1	0.3	0.5	0.6	0.8	0.1	0.3	0.5
One-hot vectors	1	0	0	0	0	0	0	0	0
	0	1	0	0	0	0	1	0	0
	0	0	1	0	0	0	0	1	0
	0	0	0	1	0	0	0	0	1
	0	0	0	0	1	0	0	0	0
	0	0	0	0	0	1	0	0	0

**Table 2:** Dataset split.

	Training	Testing	Total	Percentage
Positive	632,371	157,814	790,185	50.05
Negative	630,531	157,911	788,442	49.95
Total	1,262,902	315,725	1,578,627	
Percentage	80.00	20.00		

**Table 3:** Tested RNN architectures for the proposed solution.

Architecture	Definition
$Arch_{1}$	1 layer, 128, GRU, One-hot Vectors
$Arch_{2}$	1 layer, 128, GRU, Subunit Numbers
$Arch_{3}$	1 layer, 128, GRU, Integer Numbers
$Arch_{4}$	1 layer, 128, LSTM, One-hot Vectors
$Arch_{5}$	1 layer, 32, GRU, One-hot Vectors
$Arch_{6}$	1 layer, 64, GRU, One-hot Vectors
$Arch_{7}$	1 layer, 256, GRU, One-hot Vectors
$Arch_{8}$	2 layers, 32, GRU, One-hot Vectors
$Arch_{9}$	2 layers, 64, GRU, One-hot Vectors
$Arch_{10}$	2 layers, 128, GRU, One-hot Vectors
$Arch_{11}$	3 layers, 32, GRU, One-hot Vectors
$Arch_{12}$	3 layers, 64, GRU, One-hot Vectors
$Arch_{13}$	3 layers, 128, GRU, One-hot Vectors
$Arch_{14}$	4 layers, 32, GRU, One-hot Vectors
$Arch_{15}$	4 layers, 64, GRU, One-hot Vectors
$Arch_{16}$	5 layers, 64, GRU, One-hot Vectors
$Arch_{17}$	8 layers, 64, GRU, One-hot Vectors
$Arch_{18}$	1 layer, 128, LSTMN, One-hot Vectors
$Arch_{19}$	1 layer, 128, LSTM, One-hot Vectors, attention mechanism
$Arch_{20}$	1 layer (in each direction), 128, LSTM, One-hot Vectors,
	attention mechanism, bidirectional

**Table 4:** Results of three input preprocessing approaches on the testing dataset.

${Arch}_{i}$	Input Preprocessing	Acc (%)	PrecP (%)	PrecN (%)	Rec (%)	TNR (%)
$Arch_{1}$	One-hot Vectors	**79.81**	**78.87**	**80.81**	81.41	**78.20**
$Arch_{2}$	Subunit Numbers	74.66	73.83	75.55	76.38	72.94
$Arch_{3}$	Integer Numbers	77.87	75.53	80.67	**82.41**	73.32

**Table 5:** Results of gated recurrent unit (GRU) and long short-term memory (LSTM) experiment on the testing dataset.

${Arch}_{i}$	Memory Cells	Acc (%)	PrecP (%)	PrecN (%)	Rec (%)	TNR (%)
$Arch_{1}$	GRU	79.81	**78.87**	80.81	81.41	**78.20**
$Arch_{4}$	LSTM	**80.39**	78.56	**82.47**	**83.57**	77.21

**Table 6:** Results for varying the number of layers and number of memory cells on the testing dataset.

${Arch}_{i}$	Number of Units per Layer	Number of Layers	Acc (%)	PrecP (%)	PrecN (%)	Rec (%)	TNR (%)
$Arch_{1}$	128	1	79.81	78.87	80.81	81.41	78.20
$Arch_{5}$	32	1	78.84	78.78	78.91	78.93	78.75
$Arch_{6}$	64	1	80.34	79.53	81.20	81.70	78.99
$Arch_{7}$	256	1	77.58	76.97	78.23	78.70	76.47
$Arch_{8}$	32	2	78.62	78.74	78.50	78.39	78.85
$Arch_{9}$	64	2	**80.74**	78.52	**83.33**	**84.61**	76.87
$Arch_{10}$	128	2	78.97	79.47	78.49	78.11	**79.84**
$Arch_{11}$	32	3	79.48	79.14	79.83	80.04	78.91
$Arch_{12}$	64	3	80.67	**80.12**	81.25	81.57	79.77
$Arch_{13}$	128	3	77.84	78.05	77.65	77.47	78.22
$Arch_{14}$	32	4	80.20	79.00	81.52	82.27	78.14
$Arch_{15}$	64	4	79.92	78.53	81.44	82.32	77.51
$Arch_{16}$	64	5	80.29	79.97	80.62	80.80	79.77
$Arch_{17}$	64	8	79.96	79.52	80.42	80.69	79.23

**Table 7:** Results for varying the number of layers.

${Arch}_{i}$	Number	Acc	Acc	PrecP	PrecP	PrecN	PrecN	Rec	Rec	TNR	TNR
	of Layers	Train	Test	Train	Test	Train	Test	Train	Test	Train	Test
		(%)	(%)	(%)	(%)	(%)	(%)	(%)	(%)	(%)	(%)
$Arch_{1}$	1	84.15	**79.81**	83.36	78.87	84.99	**80.81**	85.39	**81.41**	82.90	78.20
$Arch_{10}$	2	84.87	78.97	**85.90**	**79.47**	83.90	78.49	83.50	78.11	**86.25**	**79.84**
$Arch_{13}$	3	**85.78**	77.84	84.62	78.05	**87.03**	77.65	**87.51**	77.47	84.05	78.22

**Table 8:** Results of the attention mechanism experiment (test dataset).

${Arch}_{i}$	Memory Cell	ATT	bi-RNN	Acc (%)	PrecP (%)	PrecN (%)	Rec (%)	TNR (%)
$Arch_{4}$	LSTM	no	no	**80.39**	78.56	**82.47**	**83.57**	77.21
$Arch_{18}$	LSTMN	no	no	80.34	**78.81**	82.04	82.97	**77.70**
$Arch_{19}$	LSTM	yes	no	79.42	78.06	80.93	81.83	77.01
$Arch_{20}$	LSTM	yes	yes	78.38	77.46	79.37	80.04	76.72

**Table 9:** Results of other researchers using the same dataset.

Author	Reference	Method	Training/Testing Setup	Acc (%)
Cargea et al.	[31]	CNN	Training only on this dataset	67.60
		RNN	Training only on this dataset	55.20
Chachra et al.	[33]	CNN	Not provided	80.69
Appel et al.	[32]	Hybrid	2000 tweets sample for training	86.55
Naji, I.	[4]	Naive-Bayes	Not provided	75.00
Yusuf et al.	[34]	ANN	50% train, 50% test	79.10

## Data Availability

The data used in this study is publicly available [4].
